# Supplementary material for: Precise mycobacterial species and subspecies identification using the PEP-TORCH peptidome algorithm
Source: EMBO Mol Med. 2025 Mar 4;17(4):841–61. doi: 10.1038/s44321-025-00207-5 (PMC11982334; doi:10.1038/s44321-025-00207-5)
Supplement: Supplementary file 1 — Appendix [file 44321_2025_207_MOESM1_ESM.pdf]

Appendix Supplementary Information

Precise Mycobacterial Species and Subspecies Identification Using the PEP-TORCH Peptidome Algorithm

Appendix Table S1. .... page 1
Appendix Table S2. .... page 2

Appendix Table S1.

Detailed species and peptide matching results from Unipept, corresponding to Figure 3C.

**Single Unique Peptide**			
	AQTSGNPLLTSLLN		
M. abscessus	+**		
**2-Peptide Combination**			
	AYFSTNPEAENDLR	LDTGLDKDAYQSTDFLAK	
M. abscessus	+	+	
M. chelonae	+		
M. frankinii		+	
*M. sp. H001**	+		
*M. sp. H002**	+		
*M. sp. H054**	+		
*M. sp. H072**	+		
*M. sp. H092**	+		
**3-Peptide Combination**			
	DDVSFNETLHSYGIYAPPDK	DSTDDYPVPR	GTPAQFPLGGVVPGFK
M.abcessus	+	+	+
M. franklinii	+		+
M. immunogenum		+	+
M. saopaulense	+	+	
M. salmoniphilium	+	+	
M. sp. CBMA 271	+		
M. sp. LB1	+		+
*M. sp. H001**	+	+	
*M. sp. H002**	+	+	
*M. sp. H054**	+	+	
*M. sp. H072**	+	+	
*M. sp. H092**	+	+	
*M. stephanolepidis**	+		

*, Species identified through Unipept matching but with no recorded evidence of human infection according to BV-BRC.

Appendix Table S2.
PEPTORCH Score Calculation Equation Explanation Table.

PEPTORCH Score Calculation Equation

PEPTORCH Score for each taxon (e.g., species, subspecies, or strains) based on species-specific peptide identification events recognized by the PEPTORCH algorithm. The score for each taxon is:

$$\text{Score}(a) = \frac{(E_{a1} + E_{a2} + E_{a3})}{t} \times 100\%$$

$$\text{Score}(b) = \frac{(E_{b1} + E_{b2} + E_{b3})}{t} \times 100\%$$

... ...

Where:

- **a, b**, ... : Different taxa (species, subspecies, or strains) identified by species-specific peptides.
- ***E***: An identification event triggered by single specific peptide or unique peptide combinations in the PEPTORCH algorithm for a given taxon.
- E_{a1}: The total number of single peptide identification events for taxon **a**.
- E_{a2}: The total number of two-peptide combination events for taxon **a**.
- E_{a3}: The total number of three-peptide combination events for taxon **a**.
- ***t***: Total number of identification events across ***all*** taxa ($t = \sum Ex1 + \sum Ex2 + \sum Ex3$)

Each taxon's PEPTORCH Score represents the percentage of its species peptide events relative to the total events from all taxa.

Example 1: Single infection (MAB4)

- **Taxa**: *M. abscessus* (mab)
- **Input Peptides**: 20
- **Species Peptide Events**:
  - Single-peptide events (E_{mab1}): 3
  - Two-peptide events (E_{mab2}): 61
  - Three-peptide events (E_{mab3}): 460
- **Total Events, t**:

$$t = E_{mab1} + E_{mab2} + E_{mab3} = 3 + 61 + 460 = 524$$

- **Score**:

$$\text{Score}(mab) = \frac{(3 + 61 + 460)}{524} \times 100\% = 100\%$$

- **Result**: The PEP-TORCH pipeline scores at **100%** for the unknown sample as *M. abscessus*.

Example 2: Co-infection

- **Taxa**: *M. avium* (mav) and *M. kansasii* (mkan)
- **Input Peptides**: 47
- **Species Peptide Events**:
  - For *M. avium*:
    1. (E_{mav1}): 1
    2. (E_{mav2}): 45
    3. (E_{mav3}): 994
  - For *M. kansasii*:
    1. (E_{mkan1}): 1
    2. (E_{mkan2}): 20
    3. (E_{mkan3}): 119

- **Total Events, t**:

$$\boldsymbol{t} = (E_{mab1} + E_{mav2} + E_{mav3}) + (E_{mkan1}+E_{mkan2}+E_{mkan3}) = (1 + 45 + 994) + (1 + 20 + 119) = 1180$$

- **Scores:**

$$Score(mav) = \frac{(1 + 45 + 994)}{1180} \times 100\% = 88.1\%$$

$$Score(mkan) = \frac{(1 + 20 + 119)}{1180} \times 100\% = 11.9\%$$

- **Result:** The PEP-TORCH pipeline identifies this samples as *M. avium* at 88.1% and *M. kansasii* at 11.9%.

Example 3: Subspecies Analysis (MAB3)

- **Taxa**: *M. abscessus subsp. massiliense* (submass)
- **Input Peptides**: 33
- **Subspecies Peptide Events**:
  - Single-peptide events ($E_{submass1}$): 2
  - Two-peptide events ($E_{submass2}$): 63
  - Three-peptide events ($E_{submass3}$): 961
- **Total Events, t**:

$$\boldsymbol{t} = E_{submass1} + E_{submass2} + E_{submass3} = 3 + 61 + 460 = 1026$$

- **Score**:

$$\text{Score}(submass) = \frac{(2 + 63 + 961)}{1026} \times 100\% = 100\%$$

- **Result**: The PEP-TORCH pipeline scores at **100%** for the unknown sample as *M. abscessus subsp. massiliense*.

Example 4: Subspecies Analysis (MAB22)

- **Taxa**: *M. abscessus subsp. abscessus (subab)* and *M. abscessus subsp. massiliense (submass)*
- **Input Peptides**: 33
- **Subspecies Peptide Events**:
  - For subab
    1. Single-peptide events (E_{subab1}): 2
    2. Two-peptide events (E_{subab2}): 139
    3. Three-peptide events (E_{subab3}): 4691
  - For submass
    1. Single-peptide events ($E_{submass1}$): 1
    2. Two-peptide events ($E_{submass2}$): 59
    3. Three-peptide events ($E_{submass3}$): 1711
- **Total Events, t**:

$$\boldsymbol{t} = E_{subab1} + E_{subab2} + E_{subab3} + E_{submass1} + E_{submass2} + E_{submass3} = 2+139+4691+1+59+1711 = 6603$$

- **Score**:

$$\text{Score}(subab) = \frac{(2 + 139 + 4691)}{6603} \times 100\% = 73.2\%$$

$$\text{Score}(submass) = \frac{(1 + 59 + 1711)}{6603} \times 100\% = 26.8\%$$

- **Result**: The PEP-TORCH pipeline identifies this samples as *M. abscessus subsp. abscessus* at 73.2% and *M. abscessus subsp. massiliense* at 26.8%.
