# Supplementary material for: Precise mycobacterial species and subspecies identification using the PEP-TORCH peptidome algorithm
Source: EMBO Mol Med. 2025 Mar 4;17(4):841–61. doi: 10.1038/s44321-025-00207-5 (PMC11982334; doi:10.1038/s44321-025-00207-5)
Supplement: Supplementary file 10 — Expanded View Figures [file 44321_2025_207_MOESM10_ESM.pdf]

Expanded View Figures

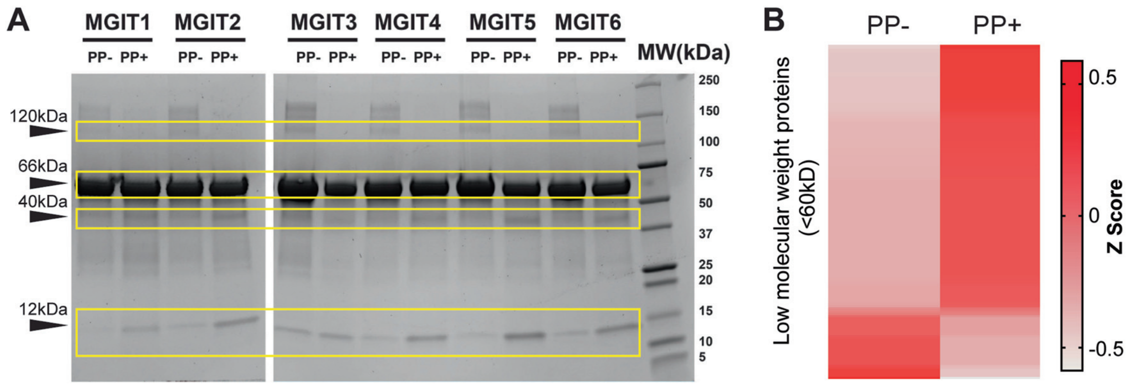

Figure EV1. Characterization of MGIT CFP samples before and after precipitation.

(**A**) SDS-PAGE analysis of protein size distributions in six MGIT CFP supernatant samples before (PP-) and after (PP+) precipitation with 50% acetonitrile, analyzed by gels stained with Coomassie blue. The 120 kDa, 66 kDa, 40 kDa, and 12 kDa protein markers are highlighted. (**B**) LC-MS/MS analysis of these six CFP samples showed at least a two-fold increase in the number of low molecular weight proteins (< 60 kDa) with enriched intensities following precipitation.

 © The Author(s)

A

Species: *M. tuberculosis*; Peptide: WDATA**T**ELN**N**ALQNL**A**R
m/z:951.4816; z = 2; RT: 75.40; -logP = 96.07, ppm = 1.2

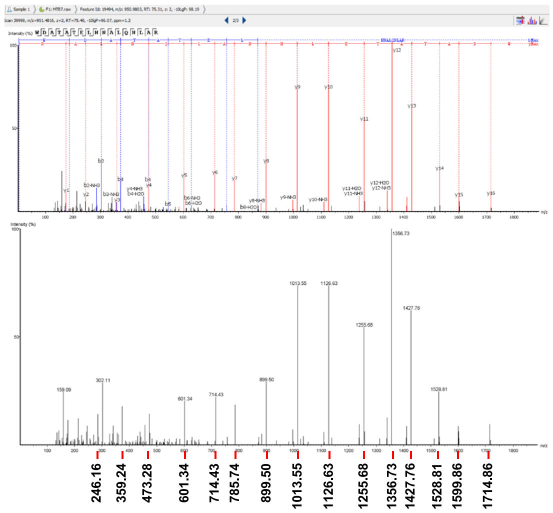

#	b	b-H2O	b-NH3	b (2+)	Seq	y	y-H2O	y-NH3	y (2+)	#
1	187.07	169.06	170.06	94.04	W					17
2	302.11	284.10	285.09	151.56	D	1714.86	1696.86	1697.84	857.94	16
3	373.15	355.14	356.12	187.07	A	1599.86	1581.83	1582.82	800.42	15
4	474.20	456.19	457.17	237.60	T	1528.81	1510.80	1511.78	764.90	14
5	545.23	527.22	528.21	273.12	A	1427.76	1409.75	1410.73	714.38	13
6	646.28	628.28	629.27	323.64	T	1356.73	1338.72	1339.71	678.86	12
7	775.33	757.32	758.31	388.16	E	1255.68	1237.66	1238.65	628.34	11
8	888.41	870.41	871.38	444.71	L	1126.63	1108.62	1109.61	563.82	10
9	1002.45	984.44	985.43	501.73	N	1013.55	995.54	996.52	507.27	9
10	1116.50	1098.49	1099.47	558.75	N	899.50	881.50	882.48	450.25	8
11	1187.53	1169.52	1170.51	594.27	A	785.47	767.45	768.44	393.23	7
12	1300.62	1282.61	1283.59	650.81	L	714.43	696.42	697.40	357.71	6
13	1428.68	1410.67	1411.65	714.84	Q	601.34	583.33	584.32	301.17	5
14	1542.72	1524.71	1525.69	771.86	N	473.28	455.26	456.26	237.14	4
15	1655.80	1637.79	1638.78	828.40	L	359.24	341.23	342.21	180.12	3
16	1726.84	1708.83	1709.81	863.92	A	246.16	228.15	229.13	123.58	2
17					R	175.12	157.11	158.09	88.06	1

Species: *M. kansasii*; Peptide: WDATA**Q**ELN**S**ALQNL**S**R
m/z:959.4832; z = 2; RT: 109.41; -logP = 88.27, ppm = 4.9

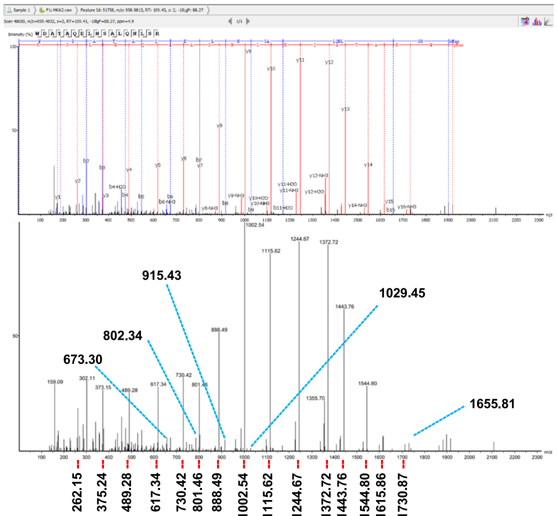

#	b	b-H2O	b-NH3	b (2+)	Seq	y	y-H2O	y-NH3	y (2+)	#
1	187.07	169.08	170.06	94.04	W					17
2	302.11	284.10	285.09	151.56	D	1730.87	1712.86	1713.85	865.93	16
3	373.15	355.14	356.13	187.07	A	1615.86	1597.83	1598.83	808.42	15
4	474.20	456.19	457.17	237.60	T	1544.80	1526.79	1527.77	772.90	14
5	545.24	527.23	528.22	273.12	A	1443.76	1425.73	1426.73	722.38	13
6	673.30	655.29	656.27	337.15	Q	1372.72	1354.71	1355.70	686.86	12
7	802.34	784.33	785.31	401.67	E	1244.67	1226.65	1227.64	622.83	11
8	915.43	897.42	898.40	458.21	L	1115.62	1097.61	1098.60	558.31	10
9	1029.45	1011.45	1012.44	515.23	N	1002.54	984.52	985.51	501.77	9
10	1116.50	1098.49	1099.47	558.75	S	888.49	870.48	871.47	444.74	8
11	1187.53	1169.52	1170.51	594.27	A	801.46	783.45	784.43	401.23	7
12	1300.62	1282.61	1283.59	650.81	L	730.42	712.41	713.40	365.71	6
13	1428.68	1410.67	1411.65	714.84	Q	617.34	599.33	600.31	309.17	5
14	1542.72	1524.71	1525.69	771.86	N	489.28	471.27	472.25	245.14	4
15	1655.81	1637.79	1638.78	828.40	L	375.24	357.22	358.21	188.12	3
16	1742.83	1724.82	1725.81	871.92	S	262.15	244.14	245.12	131.58	2
17					R	175.12	157.11	158.09	88.06	1

B

Species: *M. tuberculosis*; Peptide: TQIDQVESTA**G**SLQ**G**QWR
m/z:1002.4928; z = 2; RT: 58.67; -logP = 90.83, ppm = 0.0

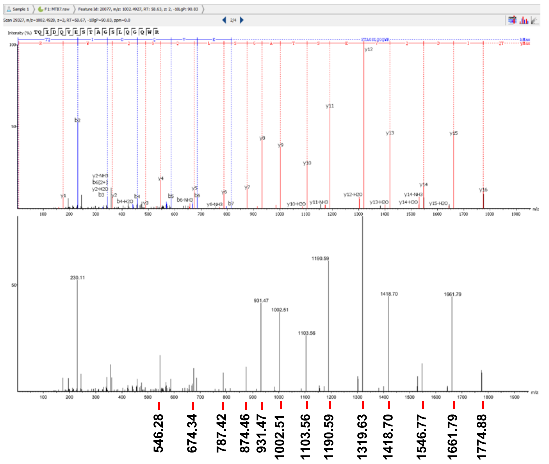

#	b	b-H2O	b-NH3	b (2+)	Seq	y	y-H2O	y-NH3	y (2+)	#
1	102.06	84.04	85.03	51.53	T					18
2	230.11	212.10	213.09	115.56	Q	1902.93	1884.92	1885.90	951.97	17
3	343.20	325.19	326.17	172.10	I	1774.88	1756.86	1757.84	887.94	16
4	458.23	440.21	441.20	229.61	D	1661.79	1643.76	1644.76	831.39	15
5	586.28	568.27	569.26	293.64	Q	1546.77	1528.75	1529.72	773.88	14
6	685.35	667.34	668.33	343.16	V	1418.70	1400.70	1401.68	709.85	13
7	814.39	796.38	797.37	407.70	E	1319.63	1301.63	1302.61	660.32	12
8	901.43	883.42	884.40	451.21	S	1190.59	1172.59	1173.58	595.80	11
9	1002.47	984.46	985.45	501.74	T	1103.56	1085.56	1086.53	552.28	10
10	1073.51	1055.50	1056.48	537.26	A	1002.51	984.49	985.49	501.76	9
11	1130.53	1112.52	1113.51	565.77	G	931.47	913.46	914.45	466.24	8
12	1217.56	1199.55	1200.54	609.28	S	874.46	856.44	857.43	437.73	7
13	1330.65	1312.64	1313.62	665.82	L	787.42	769.41	770.38	394.21	6
14	1458.71	1440.70	1441.68	729.85	Q	674.34	656.31	657.32	337.67	5
15	1515.73	1497.72	1498.70	758.36	G	546.28	528.27	529.25	273.64	4
16	1643.79	1625.78	1626.76	822.39	Q	489.26	471.25	472.23	245.13	3
17	1829.87	1811.86	1812.84	915.43	W	361.20	343.20	344.17	181.10	2
18					R	175.12	157.11	158.09	88.06	1

Species: *M. kansasii*; Peptide: TQIDQVESTA**A**SLQ**A**QWR
m/z:1017.0172; z = 2; RT: 108.27; -logP = 91.68, ppm = 7.6

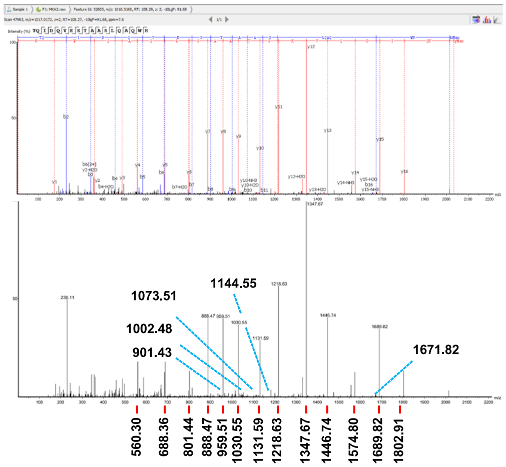

#	b	b-H2O	b-NH3	b (2+)	Seq	y	y-H2O	y-NH3	y (2+)	#
1	102.06	84.04	85.03	51.53	T					18
2	230.11	212.10	213.09	115.56	Q	1930.96	1912.95	1913.93	965.98	17
3	343.20	325.19	326.17	172.10	I	1802.91	1784.89	1785.89	901.95	16
4	458.23	440.22	441.20	229.61	D	1689.82	1671.82	1672.80	845.41	15
5	586.29	568.27	569.26	293.64	Q	1574.80	1556.78	1557.78	787.90	14
6	685.35	667.34	668.33	343.16	V	1446.74	1428.72	1429.72	723.87	13
7	814.40	796.38	797.38	407.70	E	1347.67	1329.66	1330.64	674.33	12
8	901.43	883.42	884.40	451.23	S	1218.63	1200.61	1201.61	609.81	11
9	1002.48	984.47	985.47	501.74	T	1131.59	1113.58	1114.58	566.30	10
10	1073.51	1055.50	1056.50	537.26	A	1030.55	1012.52	1013.53	515.77	9
11	1144.55	1126.54	1127.53	572.77	A	959.51	941.50	942.49	480.25	8
12	1231.58	1213.57	1214.55	616.29	S	888.47	870.46	871.45	444.73	7
13	1344.66	1326.65	1327.64	672.83	L	801.44	783.43	784.41	401.22	6
14	1472.72	1454.71	1455.70	736.86	O	688.36	670.34	671.33	344.68	5
15	1543.76	1525.75	1526.73	772.38	A	560.30	542.28	543.27	280.65	4
16	1671.82	1653.81	1654.80	836.41	Q	489.26	471.26	472.23	245.13	3
17	1857.90	1839.89	1840.87	929.45	W	361.20	343.20	344.17	181.10	2
18					R	175.12	157.11	158.09	88.06	1

© The Author(s)

◀ **Figure EV2. Shared sequences between *M. tuberculosis* and *M. kansasii.***

(**A**) The ESAT-6 peptides 'WDATATELNNALQNLAR' (left, *M. tuberculosis*) and 'WDATAQELNSALQNLSR' (right, *M. kansasii*) differ at three amino acids. (**B**) The CFP-10 peptides 'TQIDQVESTAGSLQGQWR' (left, *M. tuberculosis*) and 'TQIDQVESTAASLQAQWR' (righ, *M. kansasii*) differ at two amino acids. For each peptide, the top plot shows annotated y and b ions from PEAKS Studio, the middle plot displays the original MS2 spectrum, and the bottom table highlights distinct amino acids and corresponding ion transitions with green and blue frames.

 © The Author(s)

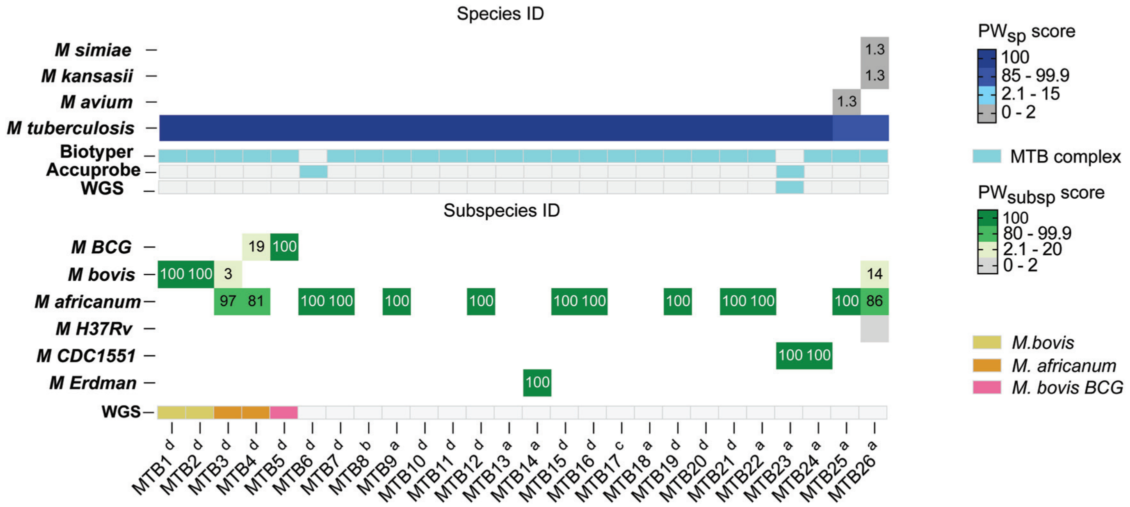

Figure EV3. *M. tuberculosis* species and subspecies output from the pipeline.

MTB species and subspecies classification using the pipeline. Samples were classified as *M. tuberculosis* or *M. tuberculosis complex* species. Nine cases, marked with an ‘a,’ were identified as *M. tuberculosis* based on peptides uniquely matching this species. Cases marked as ‘b,’ ‘c,’ and ‘d’ were classified as *M. tuberculosis* complex because the peptides identified as MTB also matched *M. canettii*, *M. orygis*, or both *M. canettii* and *M. orygis*, respectively. Subspecies identification and PWsubsp scores for each sample were determined using PEP-TORCH.

© The Author(s)

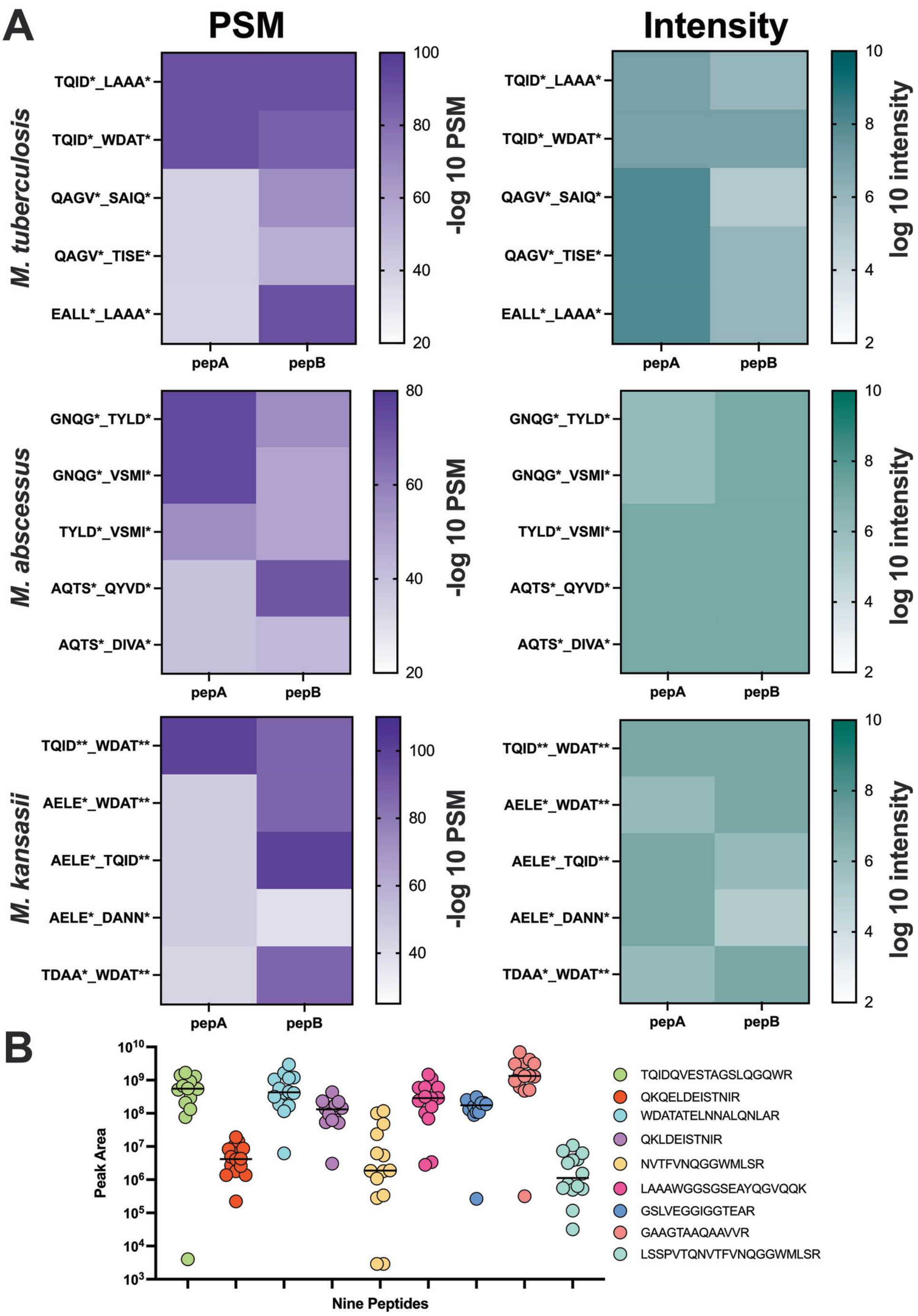

Figure EV4. Peptide-spectral match (PSM) and peak area of individual peptides in *M. tuberculosis*, *M. abscessus* and *M. kansasii* and parallel reaction monitoring (PRM) validation of nine *M. tuberculosis* target peptides.

(**A**) The median -log10 values of PSM and intensities were computed for each peptide (designated as PepA and PepB when combined) across all samples within respective species groups. Subsequently, the top five peptide combinations were identified within each species group, prioritizing those exhibiting the highest -log10 PSM and log10 intensity values. From these top combinations, three peptides were selected for further targeted analysis using PRM within each species group. The color transition from light to dark in the heat map represents the −log10 PSM values in ascending order. (**B**) All nine *M. tuberculosis*-specific peptides with high PSM scores (−log10 $P > 55$), that were validated by PRM in randomly chosen 14 samples. The blank line represents the mean peak area of the peptides which correspond to 10^6–10^8 in average.

 © The Author(s)

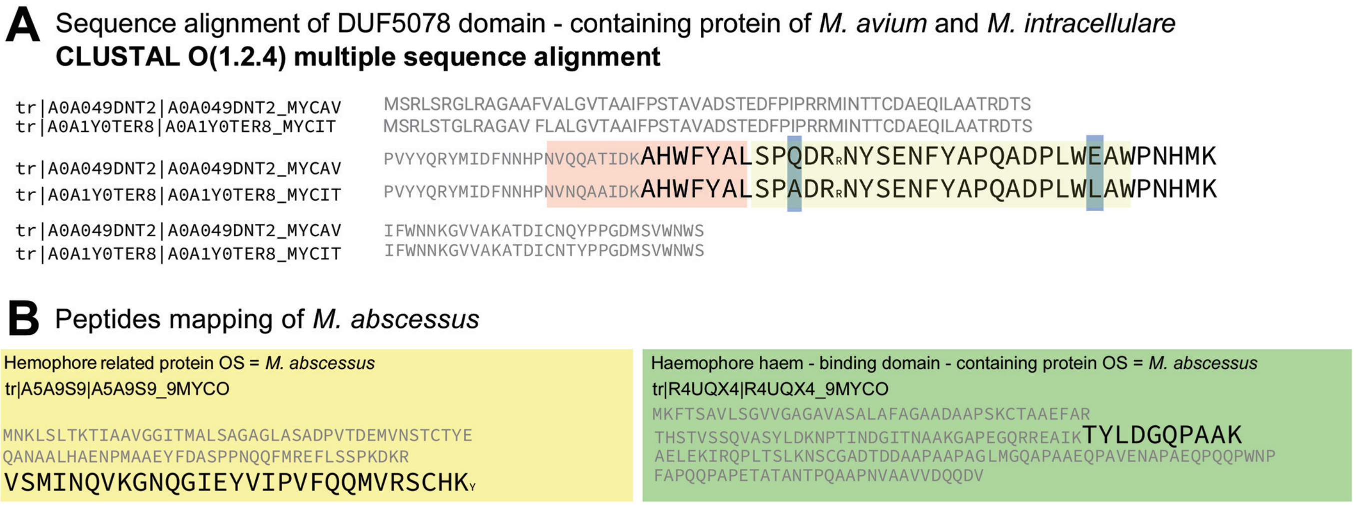

Figure EV5. Homology analysis in targeted peptides of *M. avium*, *M. intracellulare* and *M. abscessus*.

(**A**) Sequence alignment of domain-containing protein in between *M. avium* and *M. intracellulare*. Highlighted (in red and green) are the two peptide sequence regions that show single amino acid change (highlighted in blue). (**B**) Three-peptide mapping to two proteins (highlighted in yellow and green) of *M. abscessus*. The red line indicates the mapped peptide sequences.
